# Incidence and risk factors for travellers’ diarrhoea among short-term international adult travellers from high-income countries: a systematic review with meta-analysis of cohort studies

**DOI:** 10.1093/jtm/taae008

**Published:** 2024-01-15

**Authors:** Siobhan C Carroll, Maria Eugenia Castellanos, Robyn A Stevenson, Lars Henning

**Affiliations:** College of Public Health, Medical and Veterinary Sciences, James Cook University, Townsville, QLD 4811, Australia; College of Public Health, Medical and Veterinary Sciences, James Cook University, Townsville, QLD 4811, Australia; Australian Institute of Tropical Health and Medicine, James Cook University, Townsville and Cairns, QLD 4810, Australia; College of Public Health, Medical and Veterinary Sciences, James Cook University, Townsville, QLD 4811, Australia; College of Public Health, Medical and Veterinary Sciences, James Cook University, Townsville, QLD 4811, Australia

**Keywords:** Travel diarrhoea definition, gastrointestinal symptoms, pre-travel counselling, epidemiology, tourism, modified Newcastle-Ottawa Scale

## Abstract

**Introduction:**

Travellers’ diarrhoea (TD) continues to be the most common travel-related medical event in international travellers. Updated incidence and risk factor data will improve pre-travel medical advice for travellers from high-income countries (HICs), providing an opportunity for disease prevention and appropriate disease management.

**Methods:**

A systematic search for cohort studies of TD incidence published between 1 January 1997 and 2 March 2023 was performed using Ovid Medline, SCOPUS and Google Scholar databases. Study quality was assessed with a modified Newcastle-Ottawa Scale (NOS). We extracted incidence data for adults travelling less than 100 days from HIC and available risk factor data. The overall random-effects pooled incidence and the corresponding 95% confidence intervals (95% CI) were estimated. Heterogeneity was assessed using the *I*^2^ statistic, tau and the 95% prediction intervals. Subgroup analyses were conducted to identify the sources of heterogeneity. Risk factor studies were reviewed qualitatively and described.

**Results:**

Ten studies were included in the meta-analysis, containing 8478 participants. Two of the studies measured as high quality and eight as good quality as assessed by the modified NOS. The TD incidence was 36.1% (95% CI 24–41%; *I*^2^ 94%), with a prediction interval ranging from 20.3 to 55.8%. The pooled incidence of mild, moderate and severe TD was 23.6, 8.1 and 2.9%, respectively. Subgroup analysis showed that the incidence increased with increasing average data collection period. Risk factors for TD in travellers from HIC identified include younger age, longer travel periods, low and middle-income destinations, travelling for tourism, backpacking travel styles and pre-travel health status.

**Conclusion:**

It is estimated that between 20 and 56% of international travellers can expect to develop TD in travel of under 100 days. While most cases are mild, ~3% of all travellers will experience a disease that prevents usual activities or requires medical attention.

## Introduction

Travellers’ diarrhoea (TD) is a frequent occurrence in international travel, especially for tourists.^1^^,^^2^ In order for travellers and their travel health consultants to be appropriately informed about TD risk, robust incidence measurements are required. However, identifying the appropriate incidence for an individual is difficult, given the number of variables considered to contribute to TD risk, and the variability of categorization used for travel type, travel region and the definition of TD itself.^3^^,^^4^

Generally, the attack rate of TD ranges from 10 to 70% of all travellers.^5^ While the risk of a single episode of TD increases with the length of the journey, the incidence is highest during the first week and decreases thereafter.^2^^,^^6^ Actual incidence depends on traveller characteristics, the trip undertaken, the destination and their activity during the journey.

Diagnosis often relies on self-reported symptoms and is therefore a subjective assessment. Studies of incidence depend on the description provided to the cohort as to what constitutes a case of TD. In recent years, scores have been developed to advance beyond loose stool frequency.^7^^,^^8^ The three main classifications for TD have been based on (i) severity of symptoms (e.g. fever, cramps, bloody stool), (ii) number of unformed stools passed (e.g. 1–2 stool for mild, 3–5 stools for moderate and >6 stools for severe diarrhoea) and (iii) duration of symptoms (acute, persistent and chronic). Importantly, in 2017, the International Society of Travel Medicine consensus conference recommended the use of functional impairment to assess TD severity and guide TD treatment options.^9^ It has previously been found that the definition used by researchers can substantially impact the results of TD studies, with the World Health Organization (WHO) definition yielding higher case numbers, when using traditional definitions based on the frequency of stool passage.^4^

Whether tolerable or incapacitating, TD during international travel is important due to the serious chronic complications and the ramifications of incorrect treatment, including persistent abdominal symptoms, post-infectious inflammatory bowel disease and anti-microbial resistance as a result of antibiotic therapy.^10–13^ Extended spectrum beta-lactamase producing Enterobacteriales (ESBL-PE) are considered a risk to public health, and international travel has been shown to be a risk factor for ESBL-PE acquisition.^14^

Research has supported the notion that pre-travel consultations can reduce the morbidity and severity of TD.^15^ Identification of modifiable risk factors enables medical advisors to advise their clients on adaptable risk behaviours. Updated incidence and risk factor data will improve pre-travel medical advice for travellers from high-income countries (HICs), providing an opportunity for disease prevention and appropriate disease management. Predictive parameters, based on epidemiological data, are increasingly being used to guide pre-emptive treatment discussions with travellers, to minimize complications and optimize antibiotic use.^16–18^

A systematic review and meta-analysis of cohort studies was conducted to describe risk factors for TD and to provide pooled incidence estimates in adult international travellers during travel up to 100 days, based on departure from an HIC.

## Methods

A systematic literature review and meta-analysis was performed according to the Preferred Reporting Items for Systematic Reviews and Meta-Analyses Protocols (PRISMA) guidelines (Table S1, available as Supplementary data at *JTM* online).^19^^,^^20^

A systematic search was designed with the assistance of a specialist librarian and undertaken on the 20th of August 2022 using two electronic databases: Ovid Medline and SCOPUS, and one search engine: Google Scholar (Table S2, available as Supplementary data at *JTM* online). Only studies published in English after the 1st of January 1997 were included. The Google Scholar search was limited to 300, to ensure a manageable return of appropriate articles.^21^^,^^22^ The search was repeated independently by a second researcher one day later (Table S3, available as Supplementary data at *JTM* online). The same search strategy on the 2nd of March 2023, limited to 2022–23 checked for new publications (Table S4, available as Supplementary data at *JTM* online). Review of the reference lists of the retrieved articles identified no further resources. Articles were collected in an EndNote 20 library then screened for duplicates, and non-English articles, initially using EndNote functionality, followed by manual review.

The following inclusion criteria were used to ensure the review reflected the spectrum of contemporary adult travel environments: original published cohort study; the travel originated in an HIC; the travellers crossed an international border; the travel period was less than 100 days; the travellers were over 18 years of age; the cohort was not travelling for a singular purpose or shared activity (e.g. medical tourism/military deployment/cruise ship passengers), limiting the generalisability of the TD incidence; either risk factors for TD and/or TD incidence data were recorded; the data collection period began after January 1997; data collection occurred within 12 months of traveller return; TD cases did not require microbiological confirmation; and the definition of TD used to define a case was provided. In cases where the entire study did not meet the criteria, but data could be extracted which met all of the criteria, the study was included. The list of inclusion and exclusion criteria, together with the list of HIC,^23^ are available in Tables S5–S7 (available as Supplementary data at *JTM* online)*.*

**Figure 1:**
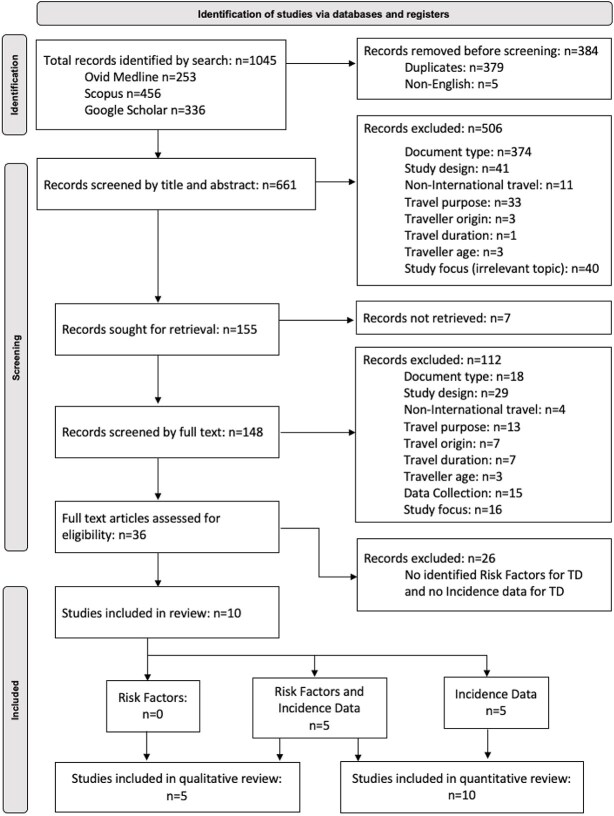
PRISMA diagram for this systematic review of incidence of and risk factors for TD

Using extraction spreadsheets created for Excel (Version 16.63.1), a total of 661 articles were included in the screening process which followed the PRISMA guidelines (Figure 1). At each stage, two researchers (S.C. and L.H.) considered the articles independently and compared outcomes. Where a consensus could not be reached, the final decision was made by a third researcher (M.E.C.) (Figures S1–S3, available as Supplementary data at *JTM* online). Ten studies met all of the inclusion criteria. All included incidence data for the quantitative synthesis. Five of those studies also included risk factor analysis appropriate for descriptive review.

A data extraction table was designed (a series of Excel Version 16.63.1 spreadsheets) to detail, categorize and analyse the contents of the selected studies (Table S8, available as Supplementary data at *JTM* online). Only published and supplementary information was included. No authors were contacted, or raw data sought. The data extraction was completed by a single researcher (S.C.) and reviewed by a second (R.S.). They met to discuss transcription errors and clarify the interpretation of data. Where interpretations differed, a third researcher (L.H.) was consulted.

The definitions of TD used in the incidence data were aligned according to the common definition closest to that described in the study; either ‘Classical diarrhoea’, ‘Individual change in bowel habits’ or ‘WHO definition of diarrhoea’ (Tables S9 and S10, available as Supplementary data at *JTM* online). Similarly, the use of the terms ‘Mild’, ‘Moderate’ and ‘Severe’ to describe the severity of TD were not uniform across the articles, so data were aligned with the closest defined category (Tables S11 and S12, available as Supplementary data at *JTM* online).

The quality of the selected studies was assessed independently by two researchers (S.C. and L.H.). The risk of bias was assessed with a modified version of the Newcastle-Ottawa Scale (NOS) for cohort studies and checked for consensus with a third researcher (M.E.C.) (Table S13, available as Supplementary data at *JTM* online).^24^ Studies were evaluated on the representativeness of participants, the comparability of the studies based on design and analysis and the adequate ascertainment of outcomes.

The meta-analysis was guided by the principles of Meta-analysis of Observational Studies in Epidemiology.^25^ Considering the variety in study design, methodologies, definitions of variables and population characteristics, heterogeneity across the studies was expected.^26^^,^^27^ Heterogeneity was assessed using *I*^2^ statistic and tau, and a random effect model was applied.^28^^,^^29^ The overall random effect pooled estimate with logit transformation was reported. As this is a meta-analysis of proportions, a prediction interval was also reported to reflect variation in true effects.^30^^,^^31^ To illustrate meta-analysis results, a forest plot showing individual and pooled estimates was generated.^26^ Outliers were identified and their effect on the overall estimate quantified using the ‘leave one out’ analyses.^32^ Overall incidence was estimated after the removal of outliers and presented as a sensitivity analysis.

The meta-analysis was performed using R software (*R Core Team (2021). R: A language and environment for statistical computing. R Foundation for Statistical Computing, Vienna, Austria.*  https://www.R-project.org/) and R studio (*RStudio Team (2020). RStudio: Integrated Development for R. RStudio, PBC, Boston, MA URL*  http://www.rstudio.com/). The *meta* and *metafor* packages were used.^33^^,^^34^

TD definition and severity were pre-specified interest groups and we estimated the incidence of TD according to definition and stratified by measure of severity. *Post hoc* subgroup analyses were undertaken according to study quality, cohort origin, cohort size, data collection period, destination, journey length, and publication date to compare the effect on overall incidence measurements and heterogeneity within the subgroups. As a meta-analysis of proportions, with noted heterogeneity, and only 10 studies, a Doi plot and Luis Furuya-Kanamori (LFK) index calculation were appropriate to visualize asymmetry and detect publication bias.^30^^,^^35^

Ethics approval was not required as this was a review of anonymised published data, available in electronic databases.

## Results

### Study characteristics

The 10 studies which met the inclusion criteria are summarized in Table 1.^36–45^ Recruitment occurred in four European and two North American countries. All of the studies recruited their participants from travel medicine clinics. One used newspaper advertising to attract participants, in addition to clinic attendees.^38^ Each study collected demographic information prior to the journey, with TD information collected after travel completion. In four studies, the travellers also recorded symptoms during the travel period.^37^^,^^41–43^

**Table 1:** Summary of studies included in this systematic review of incidence of and risk factors for TD

	Reference	Cohort used for incidence measurement	Recruitment site	Destination	Sex (% female)	Age (years)	Travel purpose	Trip duration (days)	TD definition
Incidence and risk factors	Schindler 2015	92	Switzerland	India	45.7%	*48.9% between 18 and 40*	*n/a*	*<30*	WHO
	Soonawala 2011	390	Netherlands	Tropics 24% low HDI* 56% medium HDI 20% high HDI	65%	31 (median)	61% holiday 14% VFR 13% study 8% business 4% volunteer	22.9 (mean)	Classical
	Belderok 2011	1202	Netherlands	Tropical and subtropical countries	57%	38 (median)	86% tourist 8% work/education 6% VFR	21 (mean)	Individual
	Pitzurra 2010	2800	Switzerland	22.7% SE Asia 18.6% East Africa 17% Indian subcontinent 15.5% South America	50.2%	38.8 (mean)	87.9% tourist 10.8% business 5.1% VFR	22.4 (mean)	Classical
	Stoney 2017	628	USA	26% Low HDI 51% medium HDI 23% high HDI	59%	47 (median)	67% tourist 18% business 15% volunteer 14% VFR (Could have > 1 reason)	12 (median)	Individual
Incidence only	Kuenzli 2017	171^a^	Switzerland	40.4% India, 8.4% Bhutan 22.5% Nepal 28.7% Sri Lanka	57.9%	40.9 (mean)	69.1% tourist 18.0% VFR 12.9% business	17 (median)	Individual
	Arcilla 2017	1847^b^	Netherlands	50.8% Asia 31.6% Africa 16.3% America 1.0% Europe 0.2% Oceania	54%	50.5 (median)	84.2% holiday 8.2% work 4.2% VFR 3.4% other	20 (mean)	Classical
	Vading 2016	174^c^	Sweden	38.9% SE Asia 34.9% Indian subcontinent 14.3% Northern Africa 12% Turkey	68%	49 (median)	*n/a*	14 (median)	Classical
	Lopez-Gigosos 2013	911^d^	Spain	60% Africa 40% Asia	58.4%	35 (mean)	75% tourist	*85% under 28*	Classical
	Ilnyckj 2003	109^e^	Canada	15% Mexico 7.3% India 5.8% Cuba	56%	45 (mean)	*n/a*	19 (mean)	Individual

^*^HDI = United Nations Human Development Index, n/a = Not available from published data.

^a^Participants under 18 years removed.

^b^Travellers who were ESBL positive before departure excluded from incidence data.

^c^175 in study, but TD question unanswered by 1 person.

^d^Removed participants with travel duration over 4 weeks because no maximum provided.

^e^Travellers with chronic or recent history of gastrointestinal symptoms excluded from incidence data.

The details of the quality assessment process are provided in Figure S4 (available as Supplementary data at *JTM* online). On a three-point final scale (low, good or high quality), two studies were considered high quality and the remaining eight good quality. In three studies, there was no control for TD variables, because the TD data were measured as a risk factor for another outcome. One of the studies had a large loss to follow-up without adequate explanation. No studies were low quality.

All of the studies relied on self-reporting of risk factors and outcome events, using structured questionnaires or surveys. While travel destinations were categorized differently in each study, they were generally classified as tropical and subtropical countries, with average travel duration under three weeks.

Different TD definitions were used (see Table S9, available as Supplementary data at *JTM* online). One used the WHO definition, five used ‘classical’ and four used variations of a definition reflecting individual changes in frequency or consistency of stools. While the proportion of travellers in each of the travel purpose categories varied, all seven of the studies, which provided the breakdown, showed that a majority (>60%) were ‘holiday’ or ‘tourist’ travellers.

### Systematic review

Five studies met the inclusion criteria for a review of risk factors for TD in adult short-term international travellers from HIC.^37^^,^^41–44^ Only three countries were represented in the cohort recruitment, two in Europe and one in North America.

A range of risk factors was examined across the five studies. Table 2 summarizes the risk factors considered in each study and the key findings. All of the studies examined the relationship between traveller age, sex, country of birth, duration of travel and the incidence of TD. Protective factors identified were increasing age, in two studies, and a non-western country of birth in one. Increasing duration of travel was found to be a risk factor in three studies, and female sex in one.

**Table 2:** Summary of number of cohort studies examining possible TD risk factors and the significant results reported

Variable	Number of studies	Factor found to increase risk for TD	Factor found to decrease risk for TD
*Traveller demographics*			
*Age of traveller*	5^37^^,^^41–44^		OR = 0.98, 95% CI 0.98–0.99 per unit^41^ OR = 0.38, 95% CI 0.23–0.65, over 35 years vs under^43^
*Sex of traveller*	5^37^^,^^41–44^	IRR = 1.23, 95% CI 1.04–1.46, female vs male^37^	
*Country of birth*	5^37^^,^^41–44^		IRR = 0.58, 95% CI 0.39–0.86 Non-western country of birth vs Netherlands^37^
*Trip characteristics*			
*Length of Journey*	5^37^^,^^41–44^	OR = 1.02, 95% CI 0.99–1.04, per day^43^ OR = 1.06, 95% CI 1.02–1.09, per day^44^ OR = 1.28, 95% CI 1.21–1.35, per week^41^	
*Destination*	4^37^^,^^41^^,^^43^^,^^44^	IRR = 1.94, 95% CI 1.43–2.64, South Central and Western Asia vs South America^37^ IRR = 1.93, 95% CI 1.39–2.68, Middle, Western and Northern Africa vs South America^37^ OR = 2.51, 95% CI 1.19–5.33, low-income vs high-income destinations^43^ OR = 2.29, 95% CI 1.22–4.33, medium-income vs high-income destinations^43^	
*Travel purpose*	4^37^^,^^41^^,^^43^^,^^44^		IRR = 0.63, 95% CI 0.42–0.94, VFR traveller vs tourist^37^ OR = 0.31, 95% CI 0.11–0.88, business vs holiday^43^
*Travel style*	2^42^^,^^43^	OR = 4.43 95% CI 1.25–15.75, backpacking vs luxury/middle class travel^42^	
*Accommodation type*	2^43^^,^^44^		
*Prior travel to region*	3^37^^,^^41^^,^^43^		
*Pre-travel health status*			
*Comorbidities*	2^41^^,^^42^	OR = 1.67, 95% CI 1.10–2.54, history of allergic asthma^41^	
*Medications*	3^41–43^	OR = 2.95, 95% CI 1.14–7.60, use of an antacid^43^ OR = 2.11, 95% CI 1.17–3.80, psychiatric co-medication^41^ OR = 1.38, 95% CI 1.12–1.70, malaria chemoprophylaxis^41^	
*BMI*	1^41^	OR = 1.04, 95% CI 1.01–1.08 per unit increase^41^	
*Vaccinations*	2^42^^,^^44^		
*Smoking status*	1^41^		
*Alcohol consumption*	1^41^		
*TD susceptibility*	4^41–44^		
*Pre-travel diarrhoea*	2^41^^,^^44^	OR = 2.03, 95% CI 1.59–2.54, pre-travel diarrhoea^41^	
*Behaviour and events during travel*			
*Food hygiene*	3^41^^,^^42^^,^^44^		
*New health events*	2^41^^,^^43^	OR = 6.56, 95% CI 3.06–14.04, TD-independent fever^41^ OR = 4.10, 95% CI 1.55–10.87, travel companion with TD^42^	

OR = Odds ratio, IRR = Incidence rate ratio.

Of the four studies that considered destination, two identified significant risk differences between regions. The four studies in which travel purpose was considered provided some increased risk for tourists as compared with business (one study) or visiting friends and relatives (VFR) (one study). Backpacker-style holidays were considered a risk factor in one of the two studies that included that variable.

Indicators of pre-travel health were measured in four studies. Increasing body mass index (BMI), a recent history of diarrhoea, a history of allergic asthma and the use of specific categories of medication (antacids, psychiatric medication, malaria chemoprophylaxis) were each identified as increasing risk of an episode of TD in one study. Smoking status and alcohol consumption were considered in one study, while pre-travel vaccination status was considered in two. No evidence of these as modifiable risk factors was identified.

No associations were found for food hygiene practices, prior travel to the region (three studies each) or a past history of TD (four studies). A travel companion with TD and an independent fever during the travel were both identified as risk factors in the single study which measured each of them.

### Meta-analysis

#### Overall incidence of TD

**Figure 2:**
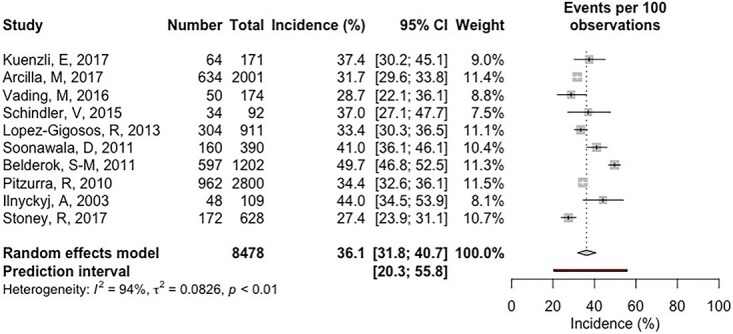
Forest plot of the cumulative TD incidence of international travellers from HICs for travel under 100 days duration

Ten studies reported the incidence of TD among 8478 short-term international travellers from HIC (Figure 2). The pooled incidence was estimated as 36.1% (95% CI 31.8, 40.7). There was considerable heterogeneity between the studies used (*I*^2^ = 94%, tau^2^ = 0.0739, *P* < 0.01). The prediction interval ranged from 20.3 to 55.8%. The leave out analyses identified one influential study, Belderok *et al.* (Figure S5, available as Supplementary data at *JTM* online). Once removed, the cumulative incidence did not vary greatly (34.3%, 95% CI 31.1, 37. *I*^2^ = 76%, tau^2^ = 0.0359, *P* < 0.01), but the prediction interval was narrower (25.5–44.6%) (Figure S6, available as Supplementary data at *JTM* online).

#### Incidence of TD according to TD definition

**Figure 3:**
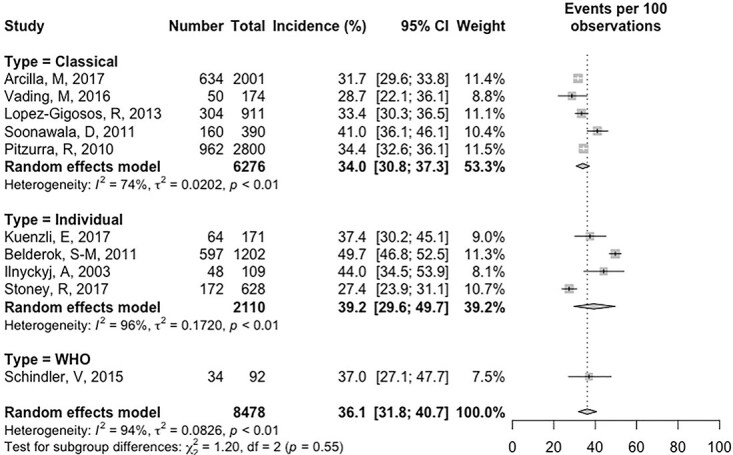
Forest plot of the TD incidence of international travellers from HICs for travel under 100 days duration stratified by TD definition

Incidence of TD did not vary based on how TD was defined, with an incidence of 34.0% (95% CI 30.8, 37.3) for the classical definition, 36.1% (95% CI 31.8, 40.7) for the WHO definition and 39.2% (95% CI 29.6, 49.7) for the individual definition (*P* = 0.55, test for subgroup differences, Figure 3).

#### Incidence of TD according to severity of the disease

**Figure 4:**
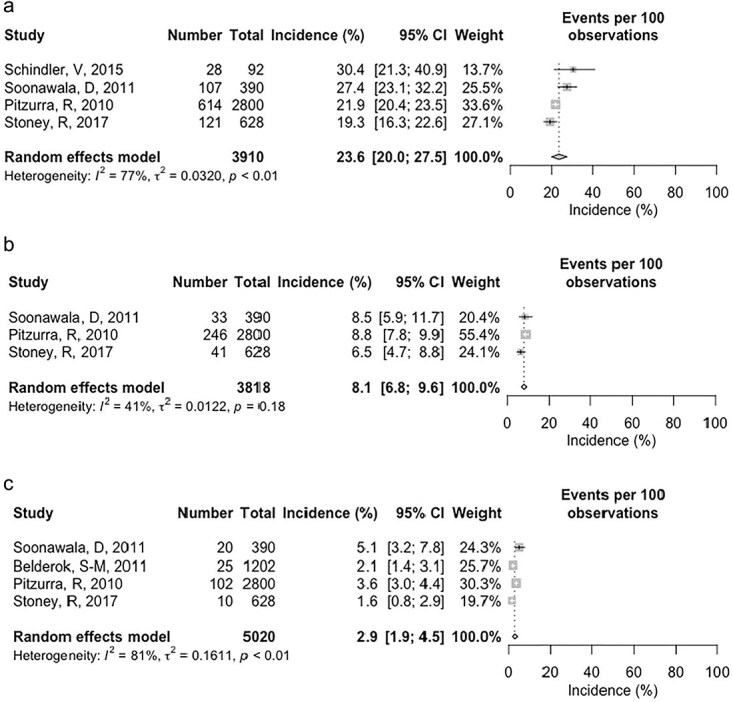
(a) Forest plot of incidence of mild TD in international travellers from HICs for travel under 100 days duration. (b) Forest plot of incidence of moderate TD in international travellers from HICs for travel under 100 days duration. (c) Forest plot of incidence of severe TD in international travellers from HICs for travel under 100 days duration

Five studies had extractable information that could be categorized to describe the severity of disease. Mild TD had a pooled incidence of 23.6% (95% CI 20.0, 27.5) from four studies (Figure 4a), moderate TD was calculated at 8.1% (95% CI 6.8, 9.6) from three studies (Figure 4b) and severe TD at 2.9% (95% CI 1.9, 4.5) from four studies (Figure 4c).

#### Post hoc subgroup analyses

Subgroup analyses by quality assessment results, cohort origin, cohort size, traveller destination and average length of journey did not explain heterogeneity and did not show differences among the subgroups (Figures S7–S10, available as Supplementary data at *JTM* online). Studies published in the last 10 years showed a lower incidence (31.5%) than studies published over 10 years ago (40.1%) (*P* = 0.02, test for subgroup differences, Figure S11, available as Supplementary data at *JTM* online). We identified that increasing average data collection period was associated with an increased reported incidence of TD, from 27.7% (95% CI 24.7, 30.9) for 2 weeks to 49.7% (95% CI 45.8, 52.5) for 4 weeks (*P* < 0.01, test for subgroup differences, Figure S12, available as Supplementary data at *JTM* online) and partially explained heterogeneity (2 weeks *I*^2^ = 0% and tau^2^ = 0; 3 weeks *I*^2^ = 79% and tau^2^ = 0.03; and unknown time *I*^2^ = 0 and tau^2^ = 0). A similar pattern of increasing incidence was found when stratifying by average journey length, with 27.7% of incidence for journeys of ≤2 weeks and 39.3% for journeys of ≤3 weeks (*P* < 0.01, test for subgroup differences, Figure S13, available as Supplementary data at *JTM* online).

#### Publication bias assessment

The Doi plot (Figure S14, available as Supplementary data at *JTM* online) with an LFK index of 0.79 did not display asymmetry that would indicate bias, particularly publication bias.

## Discussion

In this systematic review and meta-analysis of 10 cohort studies, several risk factors for TD were examined. We identified non-modifiable factors which can inform the traveller of their risk, and modifiable factors which can utilized to minimize risk where possible.

Three non-modifiable risk factors were considered in all five studies: age, sex and country of birth. The increase in risk associated with female sex identified in one of these studies has been identified in other research,^46^ but is not uniformly supported, with others showing males and females experience the same incidence of TD.^47^^,^^48^ One of the five papers identified a decreased risk in travellers from non-Western birth countries, which the authors related to the possibility of travellers returning to their region of birth, with existing immunity.^37^ Within the adult travellers considered in this review (18 years and over), increasing age was identified as protective against TD. This may not be an entirely independent finding, as age has previously been associated with higher standards of accommodation and less adventurous activities.^48–50^

This review does highlight the need for further research on the role of pre-travel health status on the risk of TD. While there was an association between certain pre-existing medical conditions, or medications, and an episode of TD, a better understanding of those relationships is needed to prove causality. In addition, further consideration of risk factors such as a travel companion with TD, which showed a strong association, would contribute to the mitigation strategies available to medical advisors during pre-travel counselling.

In terms of modifiable factors, prior research identified longer duration of travel with an increased risk of experiencing TD, and that result was repeated in our review.^2^^,^^11^ Similarly, correlation between travel to medium and low-income countries, tourist travel and backpacking have all been previously established with an increased risk of TD, and this was confirmed by the studies in our review that showed an association.^1^^,^^51^^,^^52^

In this review, compared with tourist travel, being a VFR traveller was identified as being protective against the development of TD in two studies, but no association in two others. Prior research has suggested that VFR travel could either increase or decrease the incidence of TD, depending on the destination.^53^ This illustrates the multifactorial nature of TD risk for VFR, including the importance of local sanitation and hygiene practises, social norms and risk profile.

Considering the number of variables involved, accurate calculation of TD risk relies on knowledge of individual circumstances. This meta-analysis provides a useful prediction that between 20.3 and 55.8% of international travellers from HIC will experience at least one episode of TD in travel of less than 100 days. A previous review from 2015, which included all type of studies, found that between 10 and 40% travellers develop diarrhoea.^54^ Our study has as an added value that it includes more recent studies and only includes cohort studies, providing a more unbiased and refined picture of the real burden of TD among travellers from HIC.

The incidence values were stratified according to TD definitions because they have been highlighted as a cause of variable incidence measurements in past studies.^9^ In this analysis, there was no clear difference in the measurements from the different TD definitions used, indicating that the specific wording used to define TD does not measurably alter the incidence data.

When TD incidence was grouped according to perceived severity, our results were comparable to the ratios of mild to severe disease in other original studies and reviews of TD incidence.^11^^,^^52^ The use of definitions of functional impact are extremely useful to the traveller, their medical advisor and their health insurer, in pre-travel advice and planning, as self-treatment can be explained prior to travel depending on the level of severity.^12^ While the risk of an episode of TD is significant, affecting one-third of travellers, the majority of those cases will be self-assessed as a mild inconvenience. Pre-travel advice should focus on the likelihood of a case of TD being mild and self-limiting, therefore not requiring antibiotic intervention and focus on avoiding dehydration.

The finding that 3% of travellers from HIC will experience severe TD (requiring medical attention or confinement to accommodation) is valuable in identifying cases that may lead to complicated disease. However, this group may not be homogenous. During short-term travel, the motivation to seek medical attention might be influenced by external factors rather than the severity of symptoms. For example, they may seek immediate assistance to attend a planned event or meet a scheduled departure flight. Although 8% of TD cases were self-assessed as moderate and likely self-managed, some may still benefit, for example, from antibiotic treatment.^9^ Our work does not provide guidance, however, on stand-by antibiotic use, which needs careful consideration in the individual traveller.^55^

While it is outside the scope of this review to consider the individual pathogens that are causative agents of TD, there is increasing evidence that self-administered testing or the use of predictive tools will assist in selecting appropriate treatment options according to the likely causative agent.^8^^,^^16^^,^^17^^,^^56^ Together with clinical severity classifications which consider holistic measurements beyond stool frequency, these developments will help to avoid incorrect empirical treatment, particularly in moderate and severe cases.^7^

Persistent abdominal symptoms can be among the complaints in travellers, and better understanding of the impact of negative stool results or finding a pathogen of unclear significance is needed. Diagnosis of TD is complicated because a positive laboratory result does not rule out concomitant unknown infections or necessarily prove that the identified pathogen is indeed the causative agent of symptoms.^10^ The relationship between parasitic infections and post-infectious irritable bowel syndrome remains to be fully understood, despite an increasing body of work in the area. Until now, most TD diarrhoea episodes have never received laboratory diagnosis. This might change with improved diagnostic tools based on the Whatman® FTA® technology.^11^^,^^56^

Subgroup analysis demonstrated a positive relationship between travel duration and incidence of TD. While the selection criteria ensured that data were only included from journeys under 100 days in duration, shorter average travel duration was associated with the lower values on the incidence spectrum, and vice versa. This is consistent with previous studies and the identification of travel duration as a risk factor in our systematic review.^6^ Subgroup analysis also highlighted the importance of the length of the data collection period. While this review was intended to be limited to TD cases during travel, the definition of TD can include a period of 1 week after the cessation of travel.^37^ This meta-analysis displayed an increasing TD incidence with increasing data collection period. Clinically, travellers need to understand that TD may actually occur after their return to their usual country of residence.

Our study has several limitations. By the nature of the disease, where very few cases actually seek medical care, and even fewer reach a pathological diagnosis, TD data rely on self-reporting of the event and are therefore subject to personal interpretation and bias.^4^ Additionally, every included study recruited most of their cohort from the patients presenting at pre-travel clinics. The participants may therefore be more pro-active than other travellers with regard to medical care, or planning a journey with known medical risk involved. The destination list showed a predilection for tropical and subtropical destinations, limiting the generalisability of the results to travellers visiting similar destinations.

Our study does not differentiate between viral, bacterial and protozoal causes of travel diarrhoea. More research focusing on the burden caused by specific pathogens, particularly viral pathogens, is needed. For example, a recently published prospective cohort study among travellers from Europe and the USA to areas of moderate to high risk of travel-acquired acute gastroenteritis showed that norovirus contributes to 5.2% of acute gastroenteritis cases.^57^

We note that the inconsistency of the definitions for various risk factors precludes us from quantifying the association between these factors and TD. While most studies provided data according to destination, the units used varied substantially, from geographic regions to economic index groups. Travel purpose is also categorized in a myriad of arrangements, making stratification difficult. Dupont and colleagues recently validated a newly traveller diarrhoea classification based on the multiple correspondence analysis of clinical trial data. Further studies are needed to assess its usefulness in real-life travelling scenarios.^7^

Finally, we recognize that our protocol was not registered or published before conducting our research. We developed our protocol a priori, and it is available on request from the corresponding author.

There were a number of strengths of the study. Only cohort studies were included, providing power to the risk factor associations and reliability of the incidence measurements. Very specific inclusion criteria and quality assurance measures meant that the included studies were of similar good or high quality.

## Conclusion

The findings of this study provide an updated summary for travel medicine professionals to understand and manage the risk of travellers’ diarrhoea in travellers from HIC. Pre-travel health advisors can reasonably suggest that between 20 and 56% of travellers from HIC to a tropical or subtropical destination for less than 100 days will be affected by diarrhoeal symptoms. Most of the disease is mild, with only 3% requiring confinement to bed or medical attention. Pre-travel advice regarding management of likely mild disease can prevent unnecessary use of medical services during travel, including antibiotic treatment, thereby reducing the risk of antimicrobial resistance. Health education regarding the identification of symptoms of severe disease and when to seek medical advice is warranted to prevent further complications.

## Supplementary Material

Supplementary_Information_TD_Review_09012024_taae008

## Data Availability

The full data set is available upon request from the corresponding author.

## CRediT author Statement

Siobhan Carroll (Conceptualization [equal], Data curation [equal], Investigation [lead], Methodology [equal], Project administration [lead], Software [supporting], Validation [lead], Visualization [equal], Writing—original draft [lead], Writing—review & editing [equal]), Maria Eugenia Castellanos (Conceptualization [equal], Data curation [equal], Formal analysis [equal], Methodology [equal], Software [lead], Supervision [equal], Validation [equal], Visualization [equal], Writing—review & editing [equal]), Robyn Stevenson (Data curation [supporting], Validation [supporting], Writing—original draft [supporting], Writing—review & editing [supporting]), and Lars Henning (Conceptualization [equal], Investigation [equal], Methodology [equal], Supervision [lead], Writing—review & editing [equal])
