# Supplementary material for: Incidence and risk factors for travellers’ diarrhoea among short-term international adult travellers from high-income countries: a systematic review with meta-analysis of cohort studies
Source: J Travel Med. 2024 Jan 15;32(2):taae008. doi: 10.1093/jtm/taae008 (PMC11896841; doi:10.1093/jtm/taae008)
Supplement: Supplementary_Information_TD_Review_09012024_taae008 [file supplementary_information_td_review_09012024_taae008.docx]

**List of Supplementary Tables**

Table S1 – PRISMA checklist.

Table S2 – Search terms used in this systematic review for incidence and risk factors for TD conducted on 20 August 2022 and 02 March 2023.

Table S3 – Outcome numbers from search strategy completed 20/8/2022.

Table S4 – Outcome numbers from search strategy completed 02/03/2023.

Table S5 – Explanation of exclusion criteria

Table S6 – Inclusion and Exclusion criteria used in this systematic review of incidence of and risk factors for TD.

Table S7 – List of High-Income Countries

Table S8 – Outline of data summary collection sheet

Table S9 – Definitions used in the categorisation of TD according to TD definition.

Table S10 – Categorisation of studies according to the definition of TD applied to incidence measurement used in this meta-analysis.

Table S11 – Definitions used in the classification system for functional impairment in TD to define severity, rather than stool frequency.

Table S12 – Categorisation of studies according to the severity definition applied to incidence measurement used in this meta-analysis.

Table S13 – Newcastle-Ottawa Scale for Cohort studies as modified for review of TD incidence.

Supplementary Tables

Table S1 – PRISMA Checklist

| **Section and Topic** | **Item #** | **Checklist item** | **Location where item is reported** |
| --- | --- | --- | --- |
| **TITLE** | | |  |
| Title | 1 | Identify the report as a systematic review. | Pg 1, line 5, |
| **ABSTRACT** | | |  |
| Abstract | 2 | See the PRISMA 2020 for Abstracts checklist. | Pg 2 |
| **INTRODUCTION** | | |  |
| Rationale | 3 | Describe the rationale for the review in the context of existing knowledge. | Pg 4, line 28 |
| Objectives | 4 | Provide an explicit statement of the objective(s) or question(s) the review addresses. | Pg 4, line 46 |
| **METHODS** | | |  |
| Eligibility criteria | 5 | Specify the inclusion and exclusion criteria for the review and how studies were grouped for the syntheses. | Pg 5, line 35,  Pg 46  Pg 48 |
| Information sources | 6 | Specify all databases, registers, websites, organisations, reference lists and other sources searched or consulted to identify studies. Specify the date when each source was last searched or consulted. | Pg 5, line 13 |
| Search strategy | 7 | Present the full search strategies for all databases, registers and websites, including any filters and limits used. | Pg 43 |
| Selection process | 8 | Specify the methods used to decide whether a study met the inclusion criteria of the review, including how many reviewers screened each record and each report retrieved, whether they worked independently, and if applicable, details of automation tools used in the process. | Pg 6, line 10 |
| Data collection process | 9 | Specify the methods used to collect data from reports, including how many reviewers collected data from each report, whether they worked independently, any processes for obtaining or confirming data from study investigators, and if applicable, details of automation tools used in the process. | Pg 6, line 25 |
| Data items | 10a | List and define all outcomes for which data were sought. Specify whether all results that were compatible with each outcome domain in each study were sought (e.g., for all measures, time points, analyses), and if not, the methods used to decide which results to collect. | Pg 48 |
|  | 10b | List and define all other variables for which data were sought (e.g., participant and intervention characteristics, funding sources). Describe any assumptions made about any missing or unclear information. | Pg 48  Pg 6, line 27  Pg 53, line 3  Pg 54, line 21 |
| Study risk of bias assessment | 11 | Specify the methods used to assess risk of bias in the included studies, including details of the tool(s) used, how many reviewers assessed each study and whether they worked independently, and if applicable, details of automation tools used in the process. | Pg 6, line 51 |
| Effect measures | 12 | Specify for each outcome the effect measure(s) (e.g., risk ratio, mean difference) used in the synthesis or presentation of results. | Pg 7, line 13  Pg 7, line 44 |
| Synthesis methods | 13a | Describe the processes used to decide which studies were eligible for each synthesis (e.g., tabulating the study intervention characteristics and comparing against the planned groups for each synthesis (item #5)). | Pg 6, line 25 |
|  | 13b | Describe any methods required to prepare the data for presentation or synthesis, such as handling of missing summary statistics, or data conversions. | Pg 6, line 25 |
|  | 13c | Describe any methods used to tabulate or visually display results of individual studies and syntheses. | Pg 6, line 25  Pg 7, line 21 |
|  | 13d | Describe any methods used to synthesize results and provide a rationale for the choice(s). If meta-analysis was performed, describe the model(s), method(s) to identify the presence and extent of statistical heterogeneity, and software package(s) used. | Pg 7, line 13  Pg 7, line 32 |
|  | 13e | Describe any methods used to explore possible causes of heterogeneity among study results (e.g. subgroup analysis, meta-regression). | Pg 7, line 45 |
|  | 13f | Describe any sensitivity analyses conducted to assess robustness of the synthesized results. | Pg 7, line 13 |
| Reporting bias assessment | 14 | Describe any methods used to assess risk of bias due to missing results in a synthesis (arising from reporting biases). | Pg 7, line 54 |
| Certainty assessment | 15 | Describe any methods used to assess certainty (or confidence) in the body of evidence for an outcome. | Pg 7, line 25 |
| **RESULTS** | | |  |
| Study selection | 16a | Describe the results of the search and selection process, from the number of records identified in the search to the number of studies included in the review, ideally using a flow diagram. | Pg 33  Pg 59  Pg 60 |
|  | 16b | Cite studies that might appear to meet the inclusion criteria, but which were excluded, and explain why they were excluded. | N/A |
| Study characteristics | 17 | Cite each included study and present its characteristics. | Pg 27 |
| Risk of bias in studies | 18 | Present assessments of risk of bias for each included study. | Pg 8, line 37  Pg 52 |
| Results of individual studies | 19 | For all outcomes, present, for each study: (a) summary statistics for each group (where appropriate) and (b) an effect estimate and its precision (e.g. confidence/credible interval), ideally using structured tables or plots. | Pg 29 |
| Results of syntheses | 20a | For each synthesis, briefly summarise the characteristics and risk of bias among contributing studies. | Pg 61 |
|  | 20b | Present results of all statistical syntheses conducted. If meta-analysis was done, present for each the summary estimate and its precision (e.g., confidence/credible interval) and measures of statistical heterogeneity. If comparing groups, describe the direction of the effect. | Pg 10, line 36 |
|  | 20c | Present results of all investigations of possible causes of heterogeneity among study results. | Pg 11, line 22 |
|  | 20d | Present results of all sensitivity analyses conducted to assess the robustness of the synthesized results. | Pg 10, line 43 |
| Reporting biases | 21 | Present assessments of risk of bias due to missing results (arising from reporting biases) for each synthesis assessed. | Pg 8, line 37 |
| Certainty of evidence | 22 | Present assessments of certainty (or confidence) in the body of evidence for each outcome assessed. | Pg 10, line 38 Pg 10, line 53  Pg 11, line 9 Pg 11, line 22 |
| **DISCUSSION** | | |  |
| Discussion | 23a | Provide a general interpretation of the results in the context of other evidence. | Pg 12, line 14 |
|  | 23b | Discuss any limitations of the evidence included in the review. | Pg 15, line 50 |
|  | 23c | Discuss any limitations of the review processes used. | Pg 15, line 54 |
|  | 23d | Discuss implications of the results for practice, policy, and future research. | Pg 16, line 18  Pg 17, line 13 |
| **OTHER INFORMATION** | | |  |
| Registration and protocol | 24a | Provide registration information for the review, including register name and registration number, or state that the review was not registered. | Pg 16, line 45 |
|  | 24b | Indicate where the review protocol can be accessed, or state that a protocol was not prepared. | Pg 16, line 47 |
|  | 24c | Describe and explain any amendments to information provided at registration or in the protocol. | Pg 7, line 47 |
| Support | 25 | Describe sources of financial or non-financial support for the review, and the role of the funders or sponsors in the review. | Pg 17, line 54 |
| Competing interests | 26 | Declare any competing interests of review authors. | Pg 18, line 14 |
| Availability of data, code and other materials | 27 | Report which of the following are publicly available and where they can be found: template data collection forms; data extracted from included studies; data used for all analyses; analytic code; any other materials used in the review. | Pg 18, line 10 |

*From:*  Page MJ, McKenzie JE, Bossuyt PM, *et al.* The PRISMA 2020 statement: an updated guideline for reporting systematic reviews. *BMJ* 2021;372:n71.

Table S2 - Search terms used in this systematic review for incidence and risk factors for TD conducted on 20 August 2022 and 02 March 2023.

| **Medline:** |
| --- |
| ("Travel* diarrhoea" or "travellers diarrhoea" or "travelers diarrhoea" or "travel* diarrhea" or "travelers diarrhea" or "travellers diarrhea").mp. [mp=title, book title, abstract, original title, name of substance word, subject heading word, floating sub-heading word, keyword heading word, organism supplementary concept word, protocol supplementary concept word, rare disease supplementary concept word, unique identifier, synonyms] |
| **SCOPUS:** |
| ( TITLE-ABS-KEY ( "Travel* diarrhoea"  OR  "travellers diarrhoea"  OR  "travelers diarrhoea"  OR  "travel* diarrhea"  OR  "travelers diarrhea"  OR  "travellers diarrhea" ) )  AND  ( ( TITLE-ABS-KEY ( "Risk factor*" ) )  OR  ( TITLE-ABS-KEY ( incidence ) ) ) |
| **Google scholar:** |
| ("travellers diarrhoea" OR "travelers diarrhoea" OR "travellers diarrhea" OR "travelers diarrhea") AND (“risk factors" OR "incidence”) |

Table S3 - Outcome numbers from search strategy completed 20/08/2022.
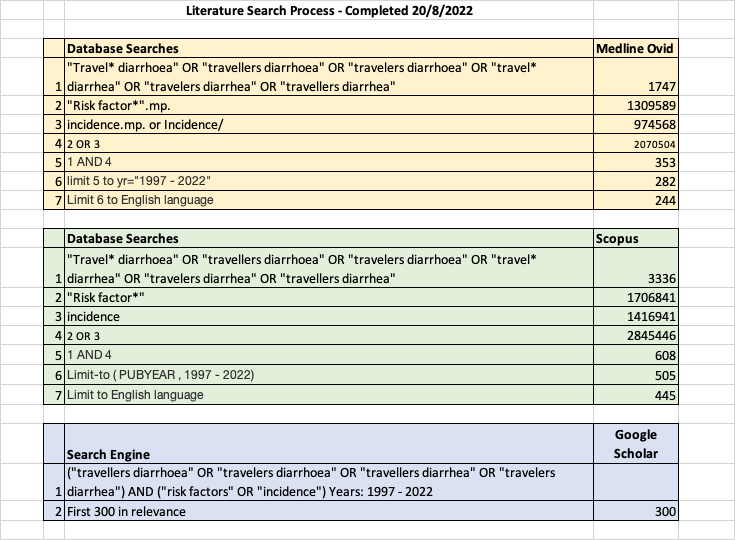


Table S4 - Outcome numbers from search strategy completed 02/03/2023.


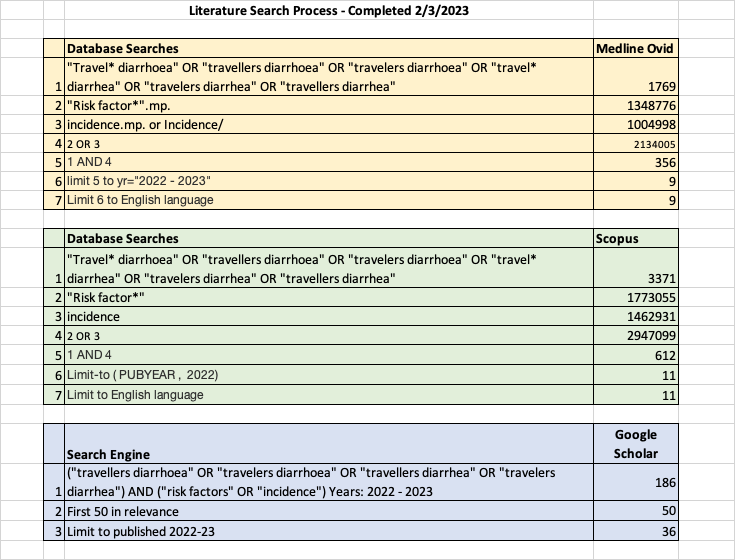


Table S5 – Explanation of exclusion criteria

| Age of data: Data collected before 1997 was considered too old to represent contemporary travel and medical environments. A 25 year period (1997-2022) was selected for limitation on the study publication date, to ensure all possible studies were included. Additionally, studies where data was collected before January 1997 were excluded. |
| --- |
| Duration of international travel: The definition of travel period was refined to a maximum period of 100 days. We considered this period most likely to capture the risk to predominantly ‘tourist-type’ travellers. |
| Age of participants: Studies recording only paediatric disease were excluded. All studies which included some travellers under the age of 18 were considered for inclusion, however, if the adult incidence data could be clearly isolated. |
| Pre-existing medical conditions: Studies where the cohort represented a specific pre-existing diagnosis, such as pregnancy or HIV-positive travellers were excluded, as they were not considered representative of an average travel cohort. |
| Country of origin (recruitment site): Studies explicitly of travellers from low or middle-income countries were excluded. High-income countries (HIC) are defined by the World Bank utilising social and economic statistics.^[[1]](#footnote-1)^ (See Table S7) |
| Definition of Travellers’ Diarrhoea: Because Travellers’ Diarrhoea has been defined differently in different settings, the term needed to be clearly defined for the study to be included. This allowed for comparison of results according to the definition used, such as Classic TD or WHO TD. Studies where the meaning of the term ‘Travellers’ Diarrhoea’ was open to interpretation by the participant, were excluded, as were studies where the definition of TD used was not provided. |
| Reason for travel (Travel purpose): The intention for travel was not always clearly stated in the articles. Studies generally included a mixture of package tourists, visitors to friends and relatives, and travel for business or professional reasons. Some studies list the main travel purpose, others allow multiple reasons, both options for recording travel purpose were included. Studies which focussed on specific, non-tourist, types of travel, such as immigration, medical tourism, and military operations, were excluded. Additionally, studies which explicitly considered a cohort with a shared activity which would negatively impact their generalisability to the experiences of an average traveller were excluded. This included studies of groups on a religious pilgrimage, cruise ship passengers, and language school students. |
| Data collection: Data collection which relied on recall over 12 months from the return date of travel were excluded, with the risk of recall bias considered too high. |
| Study Focus: To be included in the review, studies were required to have either:   1. Identified risk factors for TD, or 2. A measure of TD incidence in the study population for meta-analysis.   Therefore, studies which did not meet either of those specific requirements were excluded, and included research focussed on the treatment, microbiology, pharmacology, and long-term sequelae of travellers’ diarrhoea. |

Table S6 - Inclusion and Exclusion criteria used in this systematic review of incidence of and risk factors (RF) for TD.

| **Parameter:** | **Exclude if:** | **Include if:** |
| --- | --- | --- |
| Traveller Origin | Entire cohort is not from a High-Income Country* | Incidence or RF data can be clearly extracted for high-income group |
| Travel Duration | Cohort includes travel over 100 days | Incidence or RF data can be clearly extracted for group travelling under 100 days |
| Traveller Age | All of cohort is under 18 | Incidence or RF data can be clearly extracted for over 18 group |
| Travel Purpose | The cohort is all travelling for a specific purpose: medical tourism / military deployment / language school / cruise ship passengers | There is a mixture of travel purposes in the cohort- e.g. vacation, (VFR), business, education, other |
| Data Collection | Collection period includes time prior to January 1997  Data was collected over 12 months after traveller’s return  There was no TD data collected  Cases required microbiological confirmation | Data from within acceptable period could be isolated |
| Study Focus | No risk factors for TD and no TD incidence data | Study recorded either risk factors for TD and/or TD incidence data |
| Other | There is no definition of TD provided | Classic TD definition used  WHO TD definition used  An unusual TD definition used |

Table S7 – List of High-Income Countries

**List of High-Income Countries (HIC) ^[[2]](#footnote-2)^**

Countries and Territories classified as "high-income economies" according to the World Bank. (In brackets are the year(s) during which they held such classification; classifying began in 1987).

**High-income UN members**

Andorra (1990–present)

Antigua and Barbuda (2002, 2005–08, 2012–present)

Australia (1987–present)

Austria (1987–present)

The Bahamas (1987–present)

Bahrain (1987–89, 2001–present)

Barbados (1989, 2000, 2002, 2006–present)

Belgium (1987–present)

Brunei (1987, 1990–present)

Canada (1987–present)

Chile (2012–present)

Croatia (2008–2015, 2017–present)

Cyprus (1988–present)

Czech Republic (2006–present)

Denmark (1987–present)

Estonia (2006–present)

Finland (1987–present)

France (1987–present)

Germany (1987–present)

Greece (1996–present)

Hungary (2007–11, 2014–present)

Iceland (1987–present)

Ireland (1987–present)

Israel (1987–present)

Italy (1987–present)

Japan (1987–present)

South Korea (1993–97, 1999–present)

Kuwait (1987–present)

Latvia (2009, 2012–present)

Liechtenstein (1994–present)

Lithuania (2012–present)

Luxembourg (1987–present)

Malta (1989, 1998, 2000, 2002–present)

Monaco (1994–present)

Nauru (2015, 2019–present)

Netherlands (1987–present)

New Zealand (1987–present)

Norway (1987–present)

Oman (2007–present)

Panama (2017-2019, 2021)

Poland (2009–present)

Portugal (1994–present)

Romania (2019, 2021)

Qatar (1987–present)

Saint Kitts and Nevis (2012–present)

San Marino (1991–93, 2000–present)

Saudi Arabia (1987–89, 2003–present)

Seychelles (2014–present)

Singapore (1987–present)

Slovakia (2007–present)

Slovenia (1997–present)

Spain (1987–present)

Sweden (1987–present)

Switzerland (1987–present)

Trinidad and Tobago (2006–present)

United Arab Emirates (1987–present)

United Kingdom (1987–present)

United States (1987–present)

Uruguay (2012–present)

**High-income non-UN members**

Aruba (1987–present)

Bermuda (1987–present)

British Virgin Islands (2015–present)

Cayman Islands (1993–present)

Bailiwick of Guernsey/Jersey Channel Islands (1987–present)

Cook Islands (2016–present)

Curaçao (1994–present)a

Faroe Islands (1987–present)

French Polynesia (1990–present)

Gibraltar (2009–10, 2015–present)

Greenland (1987–present)

Guam (1987–89, 1995–present)

Hong Kong (1987–present)

Isle of Man (1987–89, 2002–present)

Macao (1994–present)

New Caledonia (1995–present)

Northern Mariana Islands (1995–2001, 2007–present)

Puerto Rico (1989, 2002–present)

Saint Martin (2010–present)

Saint Maarten (1994–present)

Taiwan (1987–present)

Turks and Caicos Islands (2009–present)

Table S8 – Outline of data summary collection sheet

| **Reference information** | PubMed ID  Authors  Title  Year of publication |
| --- | --- |
| **Study details** | Study design  Recruitment site (country)  Study period  Cohort size  Recruitment method  Measurement method  Timing of data collection  TD definition used |
| **Study population measurements**  *(Demographics,*  *Travel characteristics,*  *Possible risk factors)* | Sex  Age  Travel destination  Travel duration  Seasonality  Travel purpose (VFR/tourism/education)  Accommodation style (luxury/hostels)  Attendance at pre-travel medical consultation  Existing co-morbidities  Pre-travel vaccinations  Pre-travel medications  Unsafe food consumption practices  Any other variables described |
| **Outcome measures** | Total number of travellers with TD  Total number of episodes of TD  Severity of TD  Duration of TD episodes  Risk factors identified |
| **Additional notes** | Study value  Study weakness |

Table S9 – Definitions used in the categorisation of TD according to TD definition.

| Classical Diarrhoea | The passing of three or more unformed stools in a 24 hour period, accompanied by at least one symptom of enteric infection: nausea, faecal urgency, abdominal pain or cramps, fever, vomiting, tenesmus, or the passage of bloody/mucoid (dysenteric) stools. ^[[3]](#footnote-3)^  OR  The passage of three or more watery or loose stools per day with or without the accompanying symptoms. ^[[4]](#footnote-4)^ |
| --- | --- |
| World Health Organisation (WHO): | The passage of three or more loose or liquid stools per day (or more frequent than is normal for the individual). ^[[5]](#footnote-5)^ |
| Individual Change in Bowel Habits: | The passage of more frequent stools than is normal for the individual. Definition used in this study to consolidate those definitions which reflected the second part of the WHO definition. |

Table S10 - Categorisation of studies according to the definition of TD applied to incidence measurement used in this meta-analysis.

| **TD Incidence** | **Definition** | **References** |
| --- | --- | --- |
| **Classical diarrhoea** | 3 or more unformed stools in 24 hours with or without additional symptoms | Arcilla Lopez-Gigosos Soonawala Pitzurra |
|  | More than 3 loose stools per day during 1 or more days | Vading |
| **Individual change in bowel habits** | More frequent passage of stool than normal for the individual | Kuenzli |
|  | Diarrhea after 72 hours in your destination OR "increase in stool frequency or grade of 50% or more from baseline bowel diary" | Ilnyckyj |
|  | Self-reported diarrhea with or without nausea/vomiting, abdominal pain, or fever | Stoney |
|  | Any episode of any number of more frequent passage of loose or liquid stools per day than is normal for the individual from the beginning of the journey to the end of the first week after return | Belderok |
| **WHO definition of diarrhoea** | Classical OR Individual | Schindler |

Table S11 - Definitions used in the classification system for functional impairment in TD to define severity, rather than stool frequency. ^[[6]](#footnote-6)^

| Mild | tolerable and does not interfere with planned activities. |
| --- | --- |
| Moderate | distressing and impacts planned activities. |
| Severe | incapacitating and completely prevents planned activities. |

Table S12 - Categorisation of studies according to the severity definition applied to incidence measurement used in this meta-analysis.

| **TD Severity** | **Definition** | **References** |
| --- | --- | --- |
| **Mild** | No impact on travelling plans | Soonawala |
|  |  | Pitzurra |
|  |  | Stoney |
|  | Daily activities not impaired | Schindler |
| **Moderate** | Some influence of travelling plans | Soonawala |
|  |  | Pitzurra |
|  |  | Stoney |
| **Severe** | Causing the traveller to stay in bed or consult a medical doctor | Soonawala |
|  |  | Pitzurra |
|  |  | Stoney |
|  | Consulted a doctor | Belderok |

Table S13 – Newcastle-Ottawa Scale for Cohort studies as modified for review of TD incidence.

| NOS Component | Criterion | Option | Points |
| --- | --- | --- | --- |
| Selection | 1. Representativeness of the exposed cohort | a) representative of the average international traveller from a HIC | 2 |
|  |  | b) somewhat representative of the average international traveller from a HIC | 1 |
|  |  | c) poor/no description of the derivation of the cohort | 0 |
|  |  |  |  |
|  | 2. Cohort size | a) sample size is > 100 | 1 |
|  |  | b) sample size is <=100 | 0 |
|  |  |  |  |
|  | 3. Ascertainment of exposure / risk factors | a) medical records independently accessed by medical professional/trained person | 2 |
|  |  | b) self-reporting by structured interview/survey with medical professional/trained person | 2 |
|  |  | c) self-reporting with structured survey/questionnaire | 1 |
|  |  | d) poor/no description | 0 |
|  |  |  |  |
|  | 4. Demonstration that outcome of interest was not present at start of study | a) yes – medical records or medical assessment accessed by trained person regarding pre-existing bowel habits or disease | 2 |
|  |  | b) yes – clearly questioned regarding pre-existing bowel habits or disease | 1 |
|  |  | c) no questions regarding pre-existing bowel habits or disease | 0 |
|  |  | d) no description | 0 |
|  | Selection total | | 7 |
| Comparability | 5. Comparability of cohorts on the basis of the design or analysis | a) study controls for confounders with some effort to perform multivariate analysis | 2 |
|  |  | b) study adjusts for some co-variants | 1 |
|  |  | c) no adjustments described | 0 |
|  | Comparability total | | 2 |
| Outcome | 6. Assessment of TD | a) independent assessment by medical professional/trained person according to clear definition | 3 |
|  |  | b) self-reported according to clear definition during the journey e.g. travel diary | 2 |
|  |  | c) Self-reported according to clear definition after the journey e.g. retrospective questionnaire | 1 |
|  |  | d) Self-reported diarrhoea without clear definition | 0 |
|  |  | e) poor/no description | 0 |
|  |  |  |  |
|  | 7. Adequacy of period required to demonstrate event | a) yes – data collected at end of trip or at event | 1 |
|  |  | b) poor/no description | 0 |
|  |  |  |  |
|  | 8. Adequacy of follow-up of cohorts | a) complete follow up - all subjects accounted for | 1 |
|  |  | b) small number of subjects (<15%) lost and description provided of those lost | 1 |
|  |  | c) low follow up rate (<85%) and no/poor description of those lost | 0 |
|  |  | d) no description | 0 |
|  | Outcome total | | 5 |

Results:

**Scoring:**

0 – 5 Low quality

6 – 10 Good quality

11 – 14 High quality

|  | Range |  |
| --- | --- | --- |
| Selection | 0 - 7 |  |
| Comparability | 0 - 2 |  |
| Outcome | 0 - 5 |  |
| Total | 0 - 14 |  |

**List of Supplementary Figures**

Figure S1 – Flowchart of title and abstract screening

Figure S2 - Flowchart of full-text review

Figure S3 - Flowchart of final review against inclusion criteria

Figure S4 - Results of quality assessment according to modified Newcastle-Ottawa Scale for Travellers’ Diarrhoea Incidence and Risk Factors

Figure S5 – Results of outlier testing- Leave one out analysis.

Figure S6 – Results of outlier testing - Incidence measurement with outlier removed.

Figure S7 - Forest plot of the TD incidence of international travellers from high-income countries for travel under 100 days duration in this meta-analysis stratified by quality assessment result.

Figure S8 - Forest plot of the TD incidence of international travellers from high-income countries for travel under 100 days duration in this meta-analysis stratified by cohort origin.

Figure S9 - Forest plot of the TD incidence of international travellers from high-income countries for travel under 100 days duration in this meta-analysis stratified by cohort size.

Figure S10 - Forest plot of the TD incidence of international travellers from high-income countries for travel under 100 days duration in this meta-analysis stratified by traveller destination.

Figure S11 – Forest plot of the TD incidence of international travellers from high-income countries for travel under 100 days duration in this meta-analysis stratified by date of publication.

Figure S12 - Forest plot of the TD incidence of international travellers from high-income countries for travel under 100 days duration in this meta-analysis stratified by average data collection period.

Figure S13 - Forest plot of the TD incidence of international travellers from high-income countries for travel under 100 days duration in this meta-analysis stratified by average journey length.

Figure S14 - Doi plot of studies included in this meta-analysis of incidence of TD in international travellers from high-income countries for travel under 100 days duration.

Supplementary Figures

Figure S1 – Flowchart of title and abstract screening


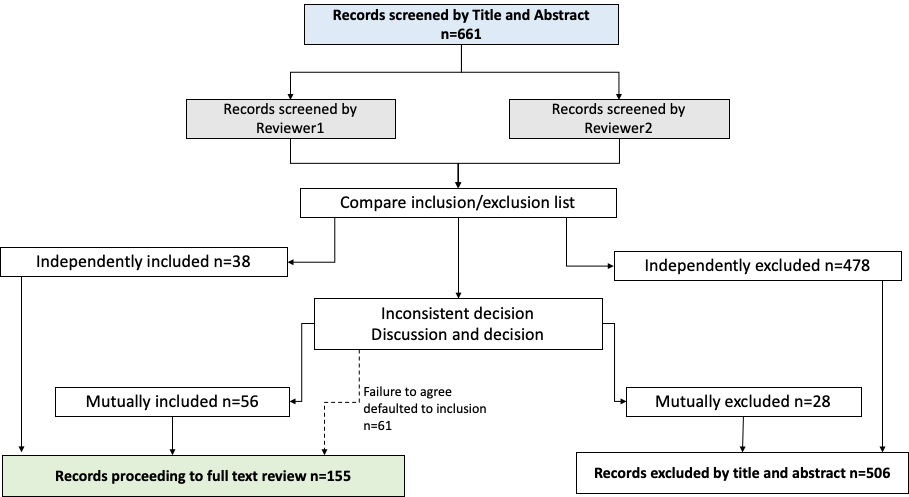


Figure S2 - Flowchart of full-text review


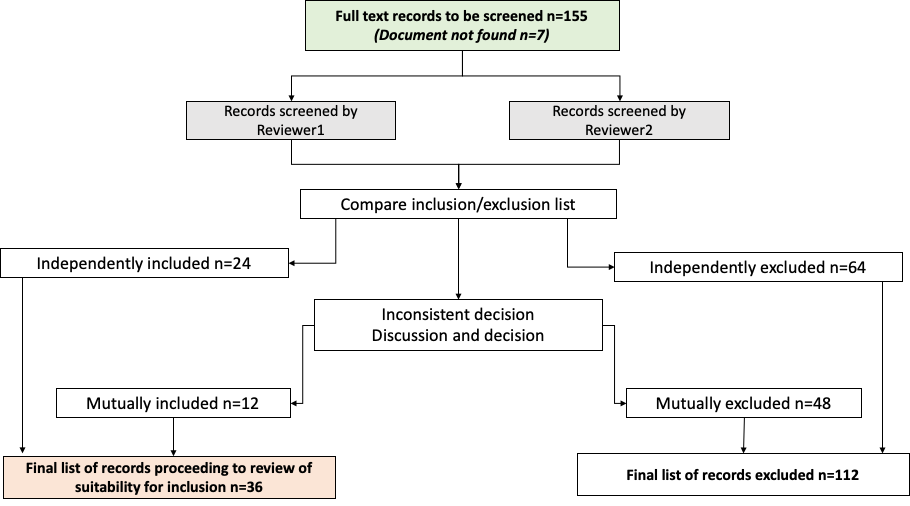


Figure S3 - Flowchart of final review against inclusion criteria

Figure S4 - Results of quality assessment according to modified Newcastle-Ottawa Scale for Travellers’ Diarrhoea Incidence and Risk Factors

| Legend | | |
| --- | --- | --- |
| Low quality | Good quality | High quality |

|  | Kuenzli, E | Arcilla, M | Vading, M | Schindler, V | Lopez-Gigosos, R | Soonawala, D | Belderok, S-M | Pitzurra, R | Ilnyckyj, A | Stoney, R |
| --- | --- | --- | --- | --- | --- | --- | --- | --- | --- | --- |
| Representativeness of cohort |  |  |  |  |  |  |  |  |  |  |
| Cohort size |  |  |  |  |  |  |  |  |  |  |
| Exposure ascertainment |  |  |  |  |  |  |  |  |  |  |
| Demonstration of new event |  |  |  |  |  |  |  |  |  |  |
| Comparability |  |  |  |  |  |  |  |  |  |  |
| Outcome assessment |  |  |  |  |  |  |  |  |  |  |
| Follow-up period |  |  |  |  |  |  |  |  |  |  |
| Completeness of follow-up |  |  |  |  |  |  |  |  |  |  |
| **Overall score** |  |  |  |  |  |  |  |  |  |  |


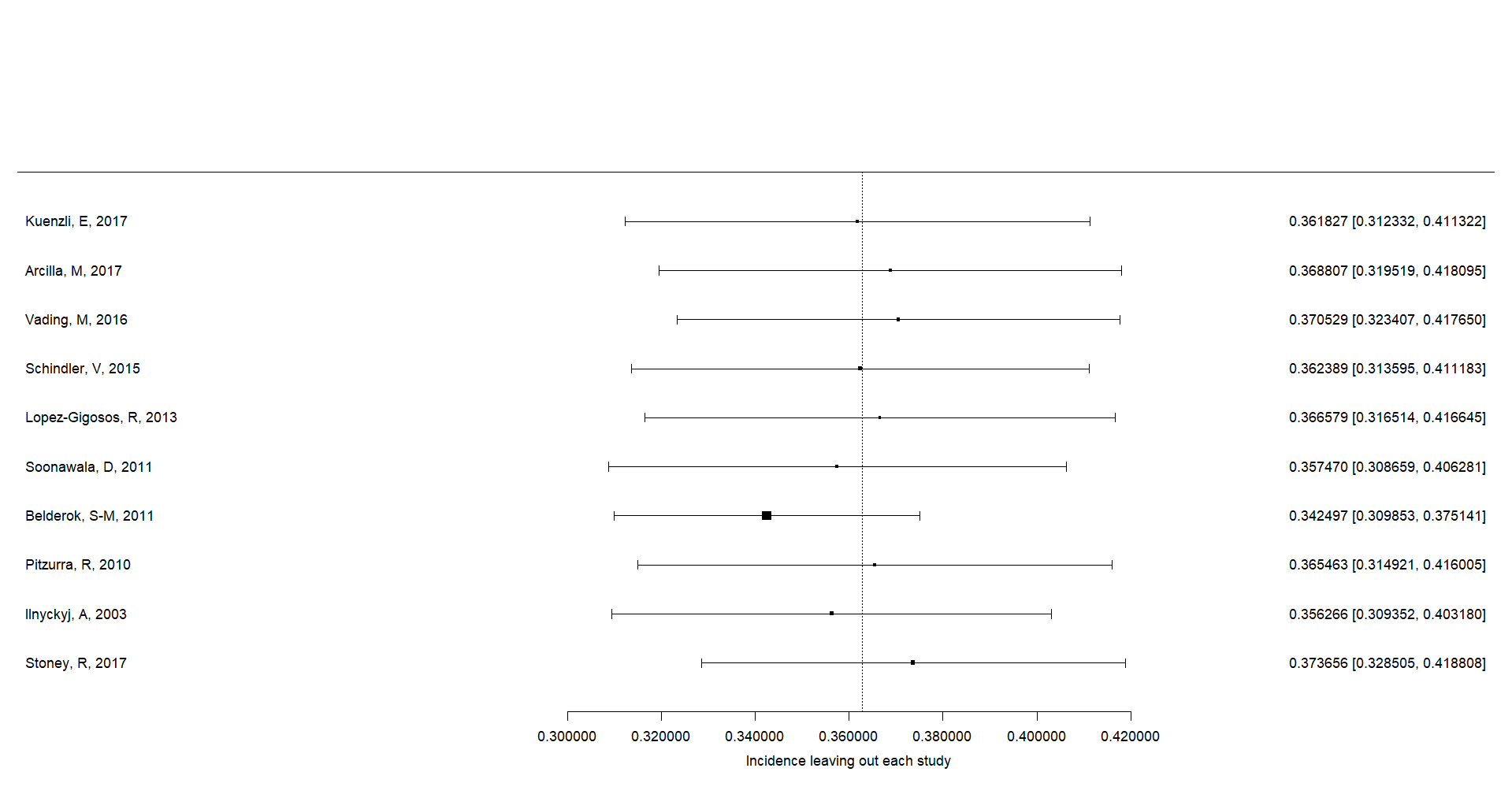
Figure S5 – Results of outlier testing- Leave one out analysis.


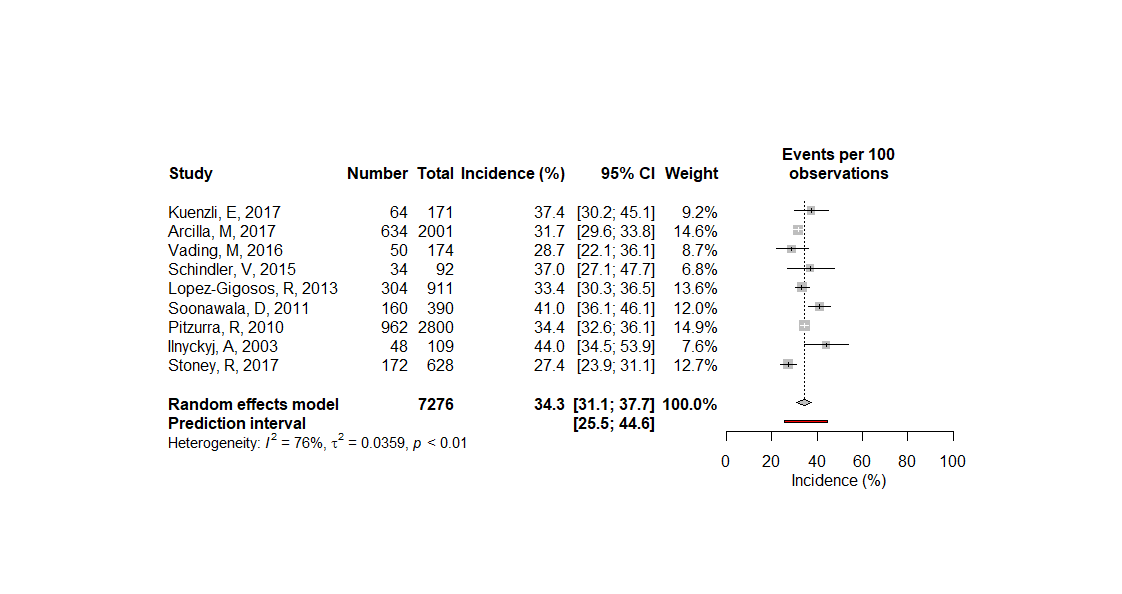
Figure S6 – Results of outlier testing - Incidence measurement with outlier removed.

Figure S7 - Forest plot of the TD incidence of international travellers from high-income countries for travel under 100 days duration in this meta-analysis stratified by quality assessment result.


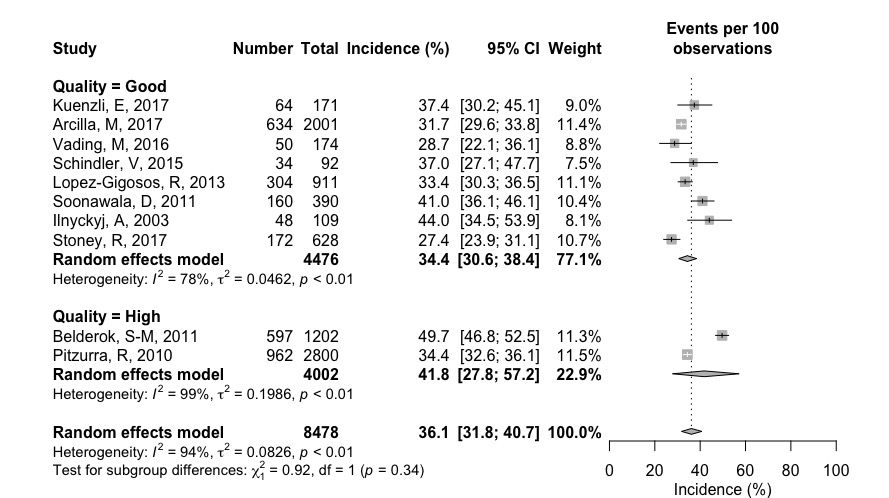


Figure S8 - Forest plot of the TD incidence of international travellers from high-income countries for travel under 100 days duration in this meta-analysis stratified by cohort origin.


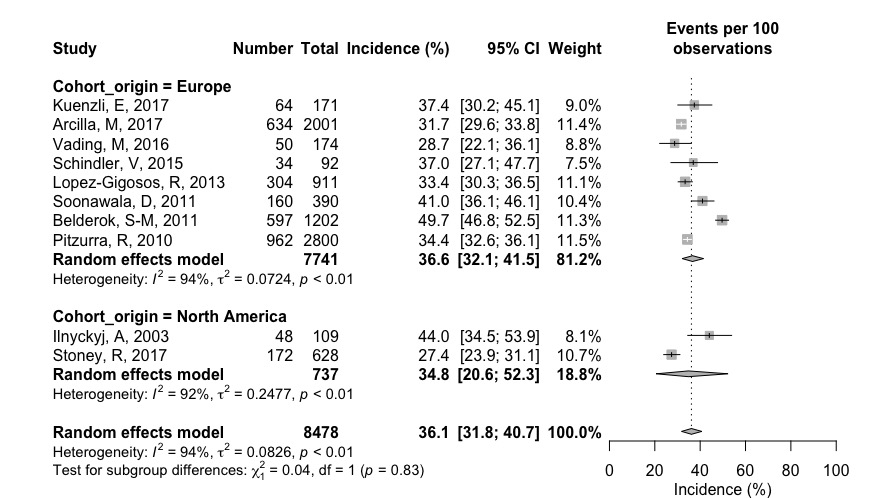


Figure S9 - Forest plot of the TD incidence of international travellers from high-income countries for travel under 100 days duration in this meta-analysis stratified by cohort size.


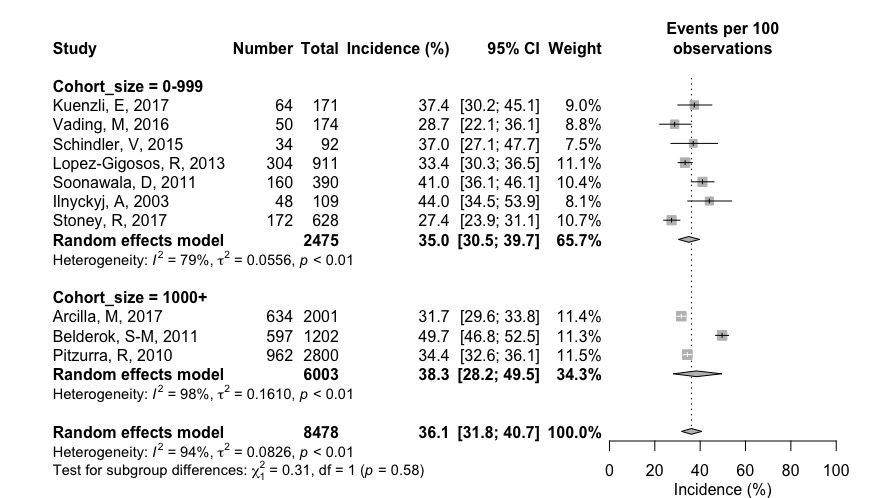


Figure S10 - Forest plot of the TD incidence of international travellers from high-income countries for travel under 100 days duration in this meta-analysis stratified by traveller destination.


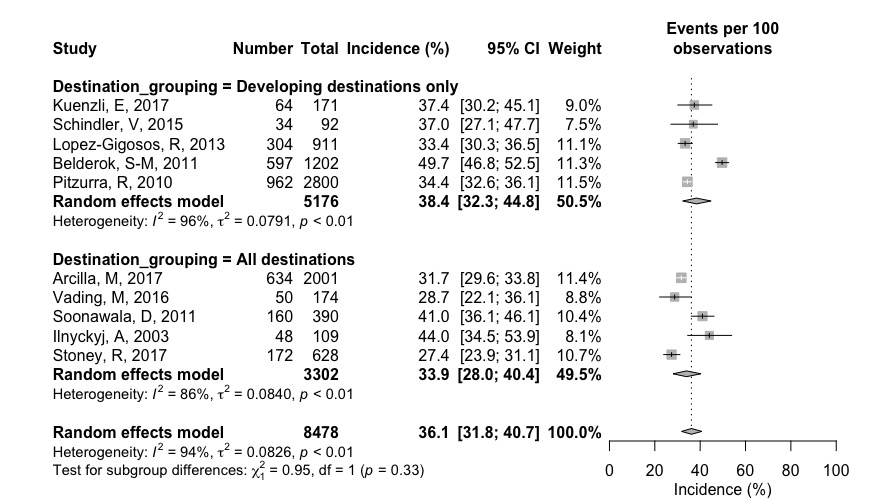


Figure S11 – Forest plot of the TD incidence of international travellers from high-income countries for travel under 100 days duration in this meta-analysis stratified by date of publication.

Figure S12 - Forest plot of the TD incidence of international travellers from high-income countries for travel under 100 days duration in this meta-analysis stratified by average data collection period.


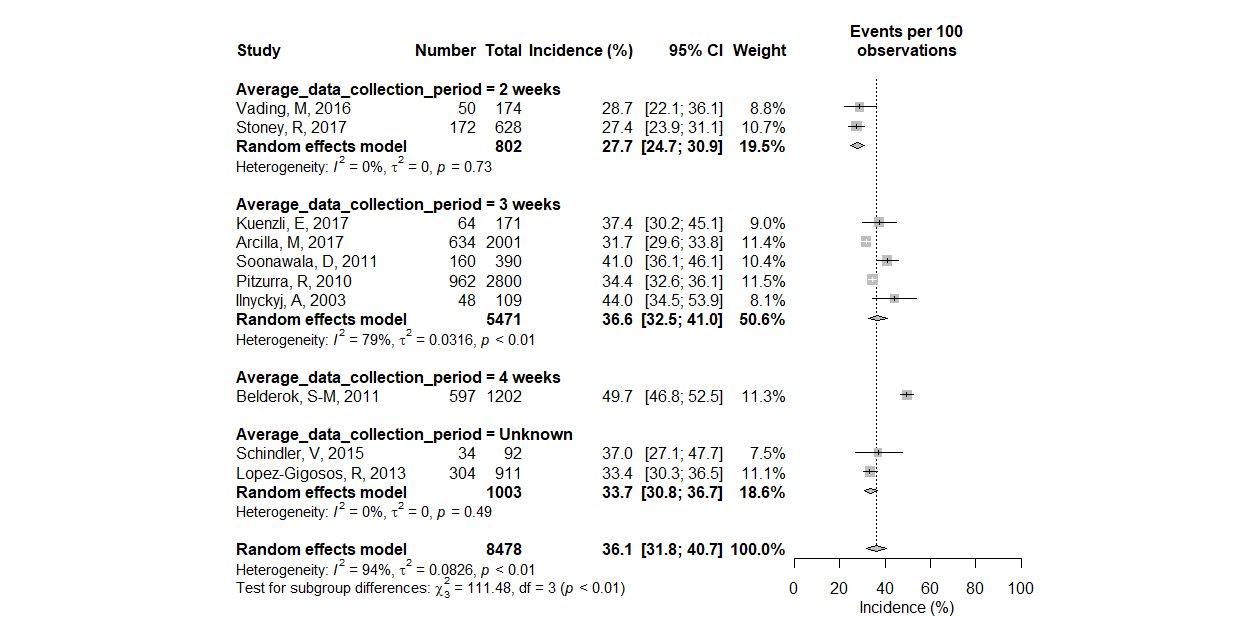


Figure S13 - Forest plot of the TD incidence of international travellers from high-income countries for travel under 100 days duration in this meta-analysis stratified by average journey length.


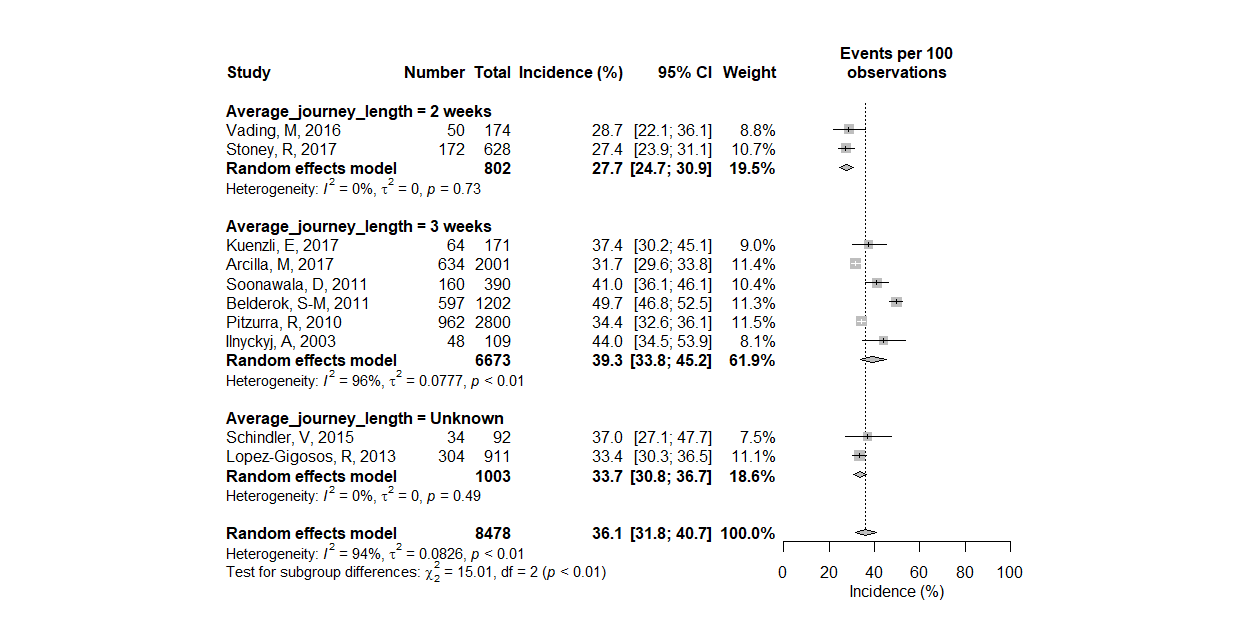


Figure S14 - Doi plot of studies included in this meta-analysis of incidence of TD in international travellers from high-income countries for travel under 100 days duration.


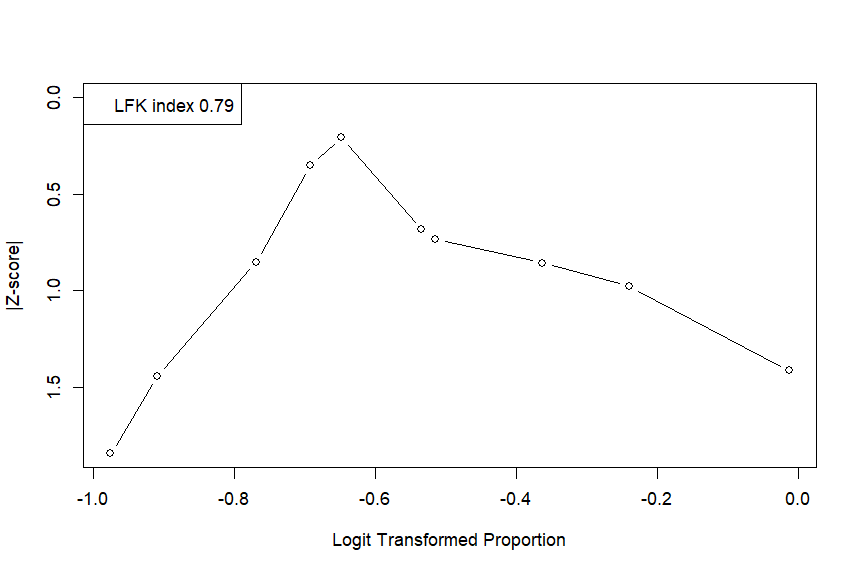


1. The World Bank. The World by Income and Region; 2022 <https://datatopics.worldbank.org/world-development-indicators/the-world-by-income-and-region.html>. Access date 30/09/2022. [↑](#footnote-ref-1)
2. The World Bank. The World by Income and Region; 2022 <https://datatopics.worldbank.org/world-development-indicators/the-world-by-income-and-region.html> Access date 30/09/2022. [↑](#footnote-ref-2)
3. Vigil KJ, DuPont HL. Travelers’ diarrhea. In: Schlossberg D, editor. *Clinical Infectious Disease (2nd ed.)* Cambridge University Press; 2015. p. 810-3. [↑](#footnote-ref-3)
4. Lääveri T, Pakkanen S, Kirveskari J, Kantele A. Travellers' diarrhoea: impact of TD definition and control group design on study results. *Travel Med Infect Dis.* 2018;24:37-43. [↑](#footnote-ref-4)
5. World Health Organisation. Diarrhoeal disease; 2017 <https://www.who.int/news-room/fact-sheets/detail/diarrhoeal-disease> Access date 14/10/22. [↑](#footnote-ref-5)
6. Riddle MS, Connor BA, Beeching NJ, *et al.* Guidelines for the prevention and treatment of travelers’ diarrhea: a graded expert panel report. *J Trav Med.* 2017;24:S63-S80. [↑](#footnote-ref-6)
